# Prevalence of Bacteremia and Bacterial Meningitis in Febrile Neonates and Infants in the Second Month of Life: A Systematic Review and Meta-analysis

**DOI:** 10.1001/jamanetworkopen.2019.0874

**Published:** 2019-03-22

**Authors:** Eric A. Biondi, Brian Lee, Shawn L. Ralston, Jared M. Winikor, Justin F. Lynn, Angela Dixon, Russell McCulloh

**Affiliations:** 1 Department of Pediatrics, Johns Hopkins Children’s Center, Baltimore, Maryland; 2 Department of Pediatrics, University of Rochester Medical Center, Rochester, New York; 3 Department of Pediatrics, Children’s Mercy Hospital, Kansas City, Missouri; 4 Department of Pediatrics, Children’s Hospital at Dartmouth, Hanover, New Hampshire; 5 Edward G. Miner Library, University of Rochester, Rochester, New York; 6 Department of Pediatrics, Children’s Hospital and Medical Center, Omaha, Nebraska

## Abstract

**Importance:**

Febrile neonates (persons in the first month of life) are believed to be at higher risk for bacteremia or bacterial meningitis than infants in their second month of life. However, the true prevalence is unclear.

**Objective:**

To determine modern rates of bacteremia and bacterial meningitis in febrile neonates and infants in the second month of life presenting to an ambulatory setting.

**Data Sources:**

A comprehensive, no-limit search was conducted in PubMed using previously published search terms in February 2015 and repeated in September 2016.

**Study Selection:**

Abstracts and full texts were reviewed independently by several investigators. Studies were included if data regarding blood cultures or cerebrospinal fluid cultures from consecutive febrile infants in an ambulatory setting could be extrapolated within the age groups. To limit the analysis to the period after the availability of the *Haemophilus influenzae* type b vaccination, studies that collected data before 1990 were excluded.

**Data Extraction and Synthesis:**

Data were extracted in accordance with the Meta-analyses of Observational Studies in Epidemiology (MOOSE) reporting guidelines via independent abstraction by several investigators. The Newcastle-Ottawa Scale was used to assess bias.

**Main Outcomes and Measures:**

The primary outcomes were prevalence rates of bacteremia and bacterial meningitis in febrile neonates and infants in the second month of life. In neonates, prevalence rates were also estimated in the era of group B *Streptococcus* intrapartum antibiotic prophylaxis (after 1996).

**Results:**

In total, 7264 abstracts were screened, resulting in 188 full-text manuscripts reviewed, with 12 meeting inclusion criteria (with 15 713 culture results). For febrile neonates, the prevalence of bacteremia was 2.9% (95% CI, 2.3%-3.7%; *I*^2^ = 50%; n = 5145) and the prevalence of bacterial meningitis was 1.2% (95% CI, 0.8%-1.9%; *I*^2^ = 27%; n = 3288). In neonates in the era after group B *Streptococcus* prophylaxis, the prevalence of bacteremia was 3.0% (95% CI, 2.3%-3.9%; *I*^2^ = 6%; n = 2055) and the prevalence of meningitis was 1.0% (95% CI, 0.4%-2.1%; *I*^2^ = 28%; n = 1739). For febrile infants in the second month of life, the prevalence of bacteremia was 1.6% (95% CI, 0.9%-2.7%; *I*^2^ = 78%; n = 4778) and the prevalence of meningitis was 0.4% (95% CI, 0.2%-1.0%; *I*^2^ = 33%; n = 2502).

**Conclusions and Relevance:**

These findings suggest that febrile neonates have approximately twice the rate of bacteremia and meningitis as febrile infants in their second month of life.

## Key Points

**Question:**

Are febrile neonates (in the first month of life) at higher risk for bacteremia and bacterial meningitis than febrile infants in their second month of life?

**Findings:**

This systematic review and meta-analysis including 15 713 culture results from 12 studies found a significant difference in the prevalence of bacteremia (2.9%) and bacterial meningitis (1.2%) in febrile neonates vs the prevalence of bacteremia (1.6%) and bacterial meningitis (0.4%) in febrile infants in their second month of life.

**Meaning:**

Febrile neonates may have roughly twice the rate of bacteremia and meningitis as febrile infants in their second month of life, although overall rates in both groups are low.

## Teaser

This systematic review and meta-analysis compares the prevalence of bacteremia and bacterial meningitis in febrile neonates vs febrile infants in their second month of life presenting to an ambulatory care setting.

## Introduction

The diagnosis and management of fever of an unknown source in infants represent a common clinical conundrum encountered by pediatricians, family medicine practitioners, and emergency medicine clinicians.^1,2^ For decades, it has been generally accepted practice that neonates (persons in the first month of life) with fever of an unknown source, given the assumption that they are at relatively high risk of bacteremia and meningitis, should undergo empirical and invasive evaluations that include laboratory work, lumbar puncture, antibiotics, and hospitalization pending the exclusion of bacterial infection via microbiological culture results.^1,2,3,4,5,6^ Infants in the second month of life who present with fever of an unknown source are generally considered to be at lower risk for bacterial infection than febrile neonates and therefore more amenable to risk stratification in their care plan.^1,3,7^

In a review on the management of fever in infants, the Agency for Healthcare Research and Quality highlighted a paucity of knowledge surrounding the stratification of risk of bacteremia or meningitis by age group.^1^ Furthermore, most commonly used risk stratification criteria were published decades ago,^5^ and in 1996, the Centers for Disease Control and Prevention published recommendations for the prevention of perinatal group B *Streptococcus* (GBS) that markedly reduced the incidence of early-onset GBS disease in neonates, one of the most common causes of bacteremia and meningitis in this age group.^8^

The primary objective of the present review was to estimate and compare the prevalence of bacteremia and meningitis in febrile neonates and infants in the second month of life. The secondary objective was to estimate and compare the prevalence of bacteremia and meningitis in febrile neonates prior to and after implementation of intrapartum GBS prophylaxis.

## Methods

The protocol for this review is registered with PROSPERO (protocol number CRD42015015996), the international prospective register of systematic reviews.^9^ This study followed the Meta-analyses of Observational Studies in Epidemiology (MOOSE) reporting guideline (eAppendix and eTable in the Supplement).^10^

### Search Strategy

A medical librarian (A.D.) conducted a comprehensive search in PubMed in February 2015 using a previously published combination of Medical Subject Headings and keywords (eAppendix in the Supplement).^1^ No limits were applied, and a total of 6792 citations were retrieved. Additional studies were identified through hand searching and manual searching of the bibliographies of qualifying studies, particularly those of other systematic reviews. Non-English articles were translated to English using translation software. Just prior to analysis, a second search was performed in September 2016 to ensure that new studies were included in the analysis. An additional 469 citations were retrieved. In the case of papers that did not meet criteria for inclusion due to lack of available data, the corresponding authors were contacted via email to determine whether additional data were available.

### Study Selection

Studies were considered eligible for inclusion if they met the following criteria: evaluated febrile infants for bacteremia or meningitis using blood cultures or cerebrospinal fluid (CSF) cultures; included consecutive febrile infants during the study date range (eg, studies identifying infants via discharge diagnosis codes were not included); included only infants seen in an ambulatory setting (ie, emergency department or outpatient clinic); allowed data abstraction within age groups representing neonates and infants in the second month of life; used blood culture or CSF culture results to determine the presence or absence of bacteremia or meningitis; provided the total number of consecutive febrile infants and the total number of those infants who had blood cultures or CSF cultures within the 2 age groups; had the intention to perform blood cultures or CSF cultures on all eligible infants in an age category (eg, a study would not be included if it was left to physician discretion whether or not to obtain CSF); and collected data in the developed world. Studies were excluded if data collection or enrollment began before 1990 to limit the analysis to the era after implementation of *Haemophilus influenzae* type b vaccination.^11^

When it was stated within a paper that it was standard policy at the study location to obtain a certain workup on all febrile infants of a certain age (eg, all febrile infants <30 days of age have CSF cultures obtained), then it was assumed that all febrile infants received that workup unless otherwise stated within the paper. Likewise, if it was stated in a paper that the standard of care was to hospitalize all febrile infants of a certain age, then the infants in that age group were included in the review even if the paper reported outcomes only for “hospitalized” infants.

### Identification and Data Extraction

All abstracts were screened by the study investigators for potential inclusion. Each full-text article from abstracts that had the potential to meet inclusion criteria was then reviewed independently by at least 2 study investigators (E.A.B., J.F.L., and R.M.), and consensus regarding inclusion was determined prior to data abstraction. For full-text articles meeting criteria, data abstraction occurred independently by 2 study investigators (E.A.B. and R.M.) who then discussed their findings to arrive at consensus. Any discrepancies were resolved by consensus of additional study investigators.

### Quality Assessment

Studies were assessed for bias using the Newcastle-Ottawa Scale (NOS) for nonrandomized studies, a tool recommended by the Cochrane Collaborative.^12^ The NOS assigns a certain number of “stars” to the potential for bias in 3 areas in each study, with more stars indicating less likelihood of bias: selection of groups (maximum 4 stars), comparability of groups (maximum 2 stars), and the determination of outcomes (maximum 3 stars). The stars can be added together to produce an overall assessment of bias. The data of interest to the present systematic review were often not the primary or secondary outcomes of the included papers; thus, the items on the scale were scored on the estimated quality of the design relative to the data of interest to this review rather than on the quality of the original study design (ie, we did not score an individual study on the basis of their original design because our population of interest and study outcome of interest may have been different from that of the original study; instead, scores were determined based on how the study included patients in our cohort of interest, whether patients with central venous catheters were excluded, etc). In addition, because the data of interest required aspects of cohort and case-control design, each study was evaluated on the item from the most applicable scale (the cohort scale was used to determine study quality regarding selection and comparability, whereas the case-control scale was used to determine study quality regarding exposure). Within the selection subsection, studies that did not obtain cultures on all infants were only awarded a maximum of 3 stars. For the comparability subsection, studies were awarded 1 star if they attempted to control for contaminants (eg, defined contaminants a priori) and a second star if they attempted to control for risk status above that of the general febrile infant population (eg, excluded infants with central venous catheters). Two study investigators (E.A.B. and R.M.) independently applied the NOS to the included studies and then discussed each score to arrive at consensus.

### Statistical Analysis

Fixed- and random-effects estimates were calculated based on inverse-variance, logit transformation of the proportion of meningitis and bacteremia cases. The exact binomial interval method was used for calculating 95% CIs, which accommodates studies in which zero events were reported. The level of heterogeneity was determined based on the *I*^2^ statistic, including corresponding 95% CIs. Given the level of heterogeneity observed, the random-effects results are reported within the text and figures. Differential summary estimates based on the cohort year were hypothesized, and consequently stratified estimates are reported. All eligible studies were included in this main analysis. To estimate potential differences in the proportions of bacteremia and meningitis cases by age group, subgroup analysis was performed for neonates in the first month of life vs infants in the second month of life. Statistical significance was defined by nonoverlapping confidence intervals.

An a priori subgroup analysis both before and after the GBS prophylaxis era was also performed for neonates, with studies reporting a data collection period that included 1996 or earlier specified as before prophylaxis, and studies reporting a data collection period beginning after 1996 specified as after prophylaxis. Two studies^3,13^ included data collected both before and after 1996, and these were designated as before prophylaxis to provide as pure a sample as possible in the modern era of GBS prophylaxis.

A subgroup analysis of the era before and after 7-valent pneumococcal conjugate vaccine (PCV7) was performed to determine whether there was a detectable change in bacteremia or meningitis rates after the introduction of the vaccine. The date of data collection for this analysis was 2001, with data collected during or after 2001 included in the after-PCV7 cohort.

An a priori sensitivity analysis was performed to assess the association with prevalence and heterogeneity by removing studies that did not attempt to control for risk. Because prevalence is reported rather than effect estimates (eg, odds ratios, risk ratios, etc), the potential for publication biases was not directly assessed; instead, the proportions and associated standard errors were relied on to address the issue. All analyses were completed using the meta package in R, version 3.3.2 (R Foundation).

## Results

**Figure 1.**
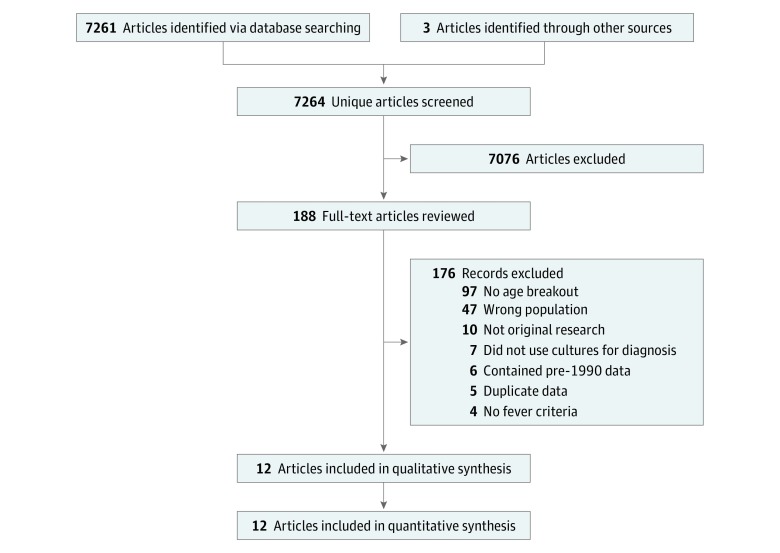
Flowchart of Eligible Studies

In total, 7264 unique articles were screened by abstract, resulting in the full-text review of 188 articles. Of these, 18 met criteria for inclusion; however, 6 of these were excluded because participants were enrolled before 1990, which left 12 studies included in the final qualitative and quantitative analysis (Figure 1). The most common reason for full-text exclusion (97 of 176 of excluded texts) was the lack of breakout of bacteremia or meningitis prevalence by age group.

### Qualitative Analysis

Of the 12 included studies, 7 took place in the United States.^3,13,14,15,16,17,18^ Eleven studies provided data for the neonatal analysis,^3,13,15,16,17,18,19,20,21,22,23^ and 5 studies provided data for older infants.^3,13,14,17,22^ Additional unpublished data from 3 papers were provided by the original authors to allow inclusion of these studies.^3,22,23^ The analyzed studies are described in the Table, including years of data collection, setting, relevant inclusion/exclusion criteria for the individual study, and outcomes pertinent to the present review.

**Table.** Description of Included Studies

Study	Date	Design	Setting	Symptoms of Population	Data on Prevalence (% of Patients)^a^	Score^b^
Bacteremia-only studies						
Chiu et al,^19^ 1994	1992-1993	P, C	ED (Taiwan)	Temperature ≥38.0°C; previously healthy^c^	0-31 d: All 254 had blood culture, 13 B (5)^d^	9
Bonsu et al,^13^ 2003	1992-1999	R, C	ED	Rectal temperature ≥38.0°C in ED or home	Study (n = 3961), 151 excluded for missing data, 1353 aged >56 d; 0-28 d: all 950 had blood culture, 14 B (2); 29-56 d: all 1507 had blood culture, 17 B (1)	7
Chiu et al,^20^ 1997	1994-1995	P, C	ED (Taiwan)	Temperature ≥38.0°C; previously healthy^c^	0-28 d: All 250 had blood culture, 11 B (4)^d^	9
Garcia et al,^22^ 2012^e^	2003-2010	R, C	ED (Spain)	Rectal temperature ≥38.0°C in ED or home	0-28 d: 207 of 307 had blood culture, 8 B (4)^d^; 29-56 d: 620 of 641 had blood culture, 21 B (3)^d^	8
				No obvious source of fever		
				No respiratory symptoms		
				No diarrhea		
Meningitis-only studies						
None						
Bacteremia and meningitis studies						
Ferrera et al,^18^ 1997	1990-1994	R, C	ED	Temperature ≥38.0°C in ED or home	0-28 d: (n = 188), 167 (89) had blood culture and 148 (79) had CSF culture, 6 B (4), 4 M (3); B/M concurrence not reported^f^	8
				No obvious infectious source		
Bonadio et al,^14^ 1993	1991-2000	P, C	ED	Rectal temperature ≥38.0°C in ED or home	28-56 d: All 534 had blood culture and CSF culture, 7 B (1), 4 M (1); no concurrent B/M	9
Bachur and Harper,^3^ 2001^e^	1993-1999	R, C	ED	Rectal temperature ≥38.0°C^c^	0-30 d: (n = 1298), 1215 (94) had blood culture and 1147 (88) had CSF culture, 26 B (2), 10 M (1); B/M concurrence not reported. 31-60 d: (n = 2104), 1866 (89) had blood culture and 1717 (82) had CSF culture, 19 B (1), 4 M (0); B/M concurrence not reported	8
Baker and Bell,^15^ 1999	1994-1996	P, C	ED	Rectal temperature ≥38.0°C^c^	3-28 d: All 254 had blood culture and CSF culture, 8 B (3), 4 M (2); 3 concurrent B/M	8
Herr et al,^17^ 2001	1999-2000	R, C	ED	Temperature ≥38.0°C^c^	0-28 d: (n = 179), 13 excluded for missing data, all remaining 166 had blood culture and CSF culture, 1B (1), 2 M (1); no concurrent B/M; 29-60 d: (n = 285), 17 excluded for missing data, all remaining 268 had blood culture and CSF culture, 6 B (2), 0 M (0)	8
Caviness et al,^16^ 2008	2001-2005	R, C	ED	Rectal temperature ≥38.0°C in ED	0-28 d: (n = 960), 893 (93) had blood culture and 874 (91) had CSF culture, 30 B (3), 13 M (1)^g^; B/M concurrence not reported	6
Zarkesh et al,^21^ 2011	2004-2009	R, C	ED (Iran)	Temperature ≥38.5°C in ED	0-28 d: (n = 253), 51 excluded for incomplete records, all remaining 202 had blood culture and CSF culture, 8 B (4), 1 M (0); 1 concurrent B/M	8
				No prior admission		
				Full term		
				No chronic disease		
				No recent antibiotics		
Ashkenazi-Hoffnung et al,^23^ 2011^e^	2005-2009	P, C	Pediatric department (Israel, unclear if ED or clinic)	Rectal temperature ≥38.0°C^c^	Study (n = 1584) of febrile infants aged ≤90 d, but only those aged 0-28 d were consecutive; 0-28 d: all 510 had blood culture and CSF culture, 12 B (2), 0 M (0)	9
				No chronic disease		
				Born >34 wk		
				No recent antibiotics		

Abbreviations: B, bacteremia; C, consecutively enrolled patients; CSF, cerebrospinal fluid; ED, emergency department; M, meningitis; P, prospective; R, retrospective.

^a^ Percentages are rounded to nearest integer.

^b^ Newcastle-Ottawa Scale bias scores range from 1 (worst) to 9 (best).

^c^ Unclear whether this status needed to be during evaluation or if it could have been at home as well.

^d^ Unable to use CSF culture data owing to inability to identify the total number of CSF cultures obtained or owing to potential selection bias (eg, CSF culture obtained only if clinical suspicion for meningitis).

^e^ Additional unpublished data obtained via correspondence with original authors.

^f^ There were 724 infants presenting in the ED; 43 incomplete records were excluded; of the remaining 681 infants, 188 had fever without an obvious source, 21 had undocumented blood culture, and 40 had undocumented CSF culture.

^g^ There may have been 12 of 874 (1%); unclear owing to rounding in the fluid cultures of the original study.

Overall NOS scores ranged from 7 to 9 (maximum score, 9) for the 12 included studies (Table). Scores within individual categories for each study are provided in the eTable in the Supplement.

### Quantitative Analysis

**Figure 2.**
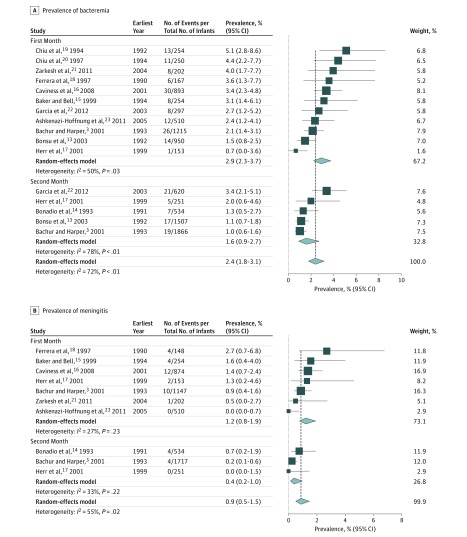
Meta-analysis of Prevalence of Bacteremia and Meningitis in Febrile Neonates and Infants in the First and Second Months of Life For each subgroup, the sum of the statistics, along with the summary prevalence, is represented by the middle of the solid diamonds. The width of the diamonds represents summary 95% CIs; squares represent mean values, with error bars representing 95% CIs.

Data from 15 713 blood cultures and CSF cultures were included in this analysis. Analysis of bacteremia prevalence for all neonates and infants in the second month of life included 9923 children from 12 studies. Random-effects modeling estimated the prevalence of bacteremia in the first 2 months of life to be 2.4% (95% CI, 1.8%-3.1%; *I*^2^ = 72%) (Figure 2A). The meningitis analysis for the first 2 months of life included 5790 infants from 8 studies. Random-effects modeling estimated the prevalence of bacterial meningitis in the first 2 months of life to be 0.9% (95% CI, 0.5%-1.5%; *I*^2^ = 55%) (Figure 2B).

**Figure 3.**
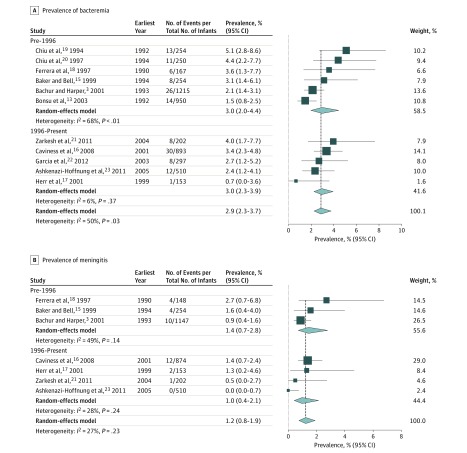
Meta-analysis of Prevalence of Bacteremia and Meningitis in Febrile Neonates in the First Month of Life For each subgroup, the sum of the statistics, along with the summary prevalence, is represented by the middle of the solid diamonds. The width of the diamonds represents summary 95% CIs; squares represent mean values, with error bars representing 95% CIs.

The bacteremia analysis for the first month of life included 5145 neonates from 11 studies. Five of these studies (2055 neonates) collected all data after 1996. Random-effects modeling of all studies estimated the prevalence of bacteremia in febrile neonates to be 2.9% (95% CI, 2.3%-3.7%; *I*^2^ = 50%) (Figure 3A). When only studies containing data from the GBS prophylaxis era (after 1996) were analyzed, the rate of bacteremia remained 3.0% (95% CI, 2.3%-3.9%; *I*^2^ = 6%) (Figure 3A).

The meningitis analysis for the first month of life included 3288 neonates from 7 studies. Four of these studies (1739 neonates) collected all data after 1996. Random-effects modeling of all studies estimated the prevalence of meningitis in febrile neonates to be 1.2% (95% CI, 0.8%-1.9%; *I*^2^ = 27%) (Figure 3B). When only studies containing data from the GBS prophylaxis era (after 1996) were included, the prevalence of meningitis was 1.0% (95% CI, 0.4%-2.1%; *I*^2^ = 28%) (Figure 3B).

**Figure 4.**
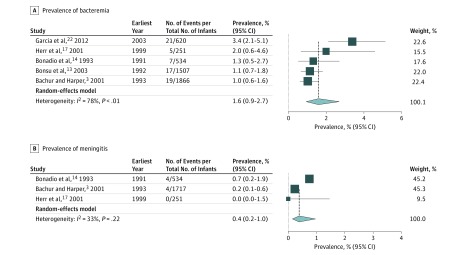
Meta-analysis of Prevalence of Bacteremia and Meningitis in Febrile Infants in the Second Month of Life For each subgroup, the sum of the statistics, along with the summary prevalence, is represented by the middle of the solid diamonds. The width of the diamonds represents summary 95% CIs; squares represent mean values, with error bars representing 95% CIs.

The bacteremia analysis for the second month of life included 4778 infants from 5 studies. Random-effects modeling estimated the prevalence of bacteremia in febrile infants in the second month of life to be 1.6% (95% CI, 0.9%-2.7%; *I*^2^ = 78%) (Figure 4A).

The bacterial meningitis analysis for the second month of life included 2502 infants from 3 studies. Random-effects modeling estimated the prevalence of bacterial meningitis in febrile infants in the second month of life to be 0.4% (95% CI, 0.2%-1.0%; *I*^2^ = 33%) (Figure 4B).

Our before- and after-PCV7 analysis did not identify statistically significant before and after differences in rates of bacteremia in the first month of life (95% CI, 1.9%-4.2% [*I*^2^ = 66%] vs 95% CI, 2.4%-4.0% [*I*^2^ = 0%], respectively) or meningitis in the first month of life (95% CI, 0.8%-2.3% [*I*^2^ = 24%] vs 95% CI, 0.2%-2.5% [*I*^2^ = 52%], respectively). For bacteremia in the second month of life, there was only 1 study performed in the period after PCV7, and for meningitis in the second month, none were performed.

### Sensitivity Analyses

The sensitivity analysis removing studies that did not attempt to control for risk above that of the general population of febrile neonates resulted in a bacteremia prevalence estimate of 3.1% (95% CI, 2.3%-4.2%; *I*^2^ = 44%; n = 3048) and a bacterial meningitis prevalence estimate of 1.0% (95% CI, 0.5%-2.2%; *I*^2^ = 46%; n = 2160) for neonates. These estimates were not significantly different from the full analysis or the after-GBS prophylaxis era analysis. The sensitivity analysis of febrile infants in the second month of life, removing studies that did not attempt to control for risk above that of the general population of febrile infants, resulted in a bacteremia prevalence estimate of 1.7% (95% CI, 0.9%-3.4%; *I*^2^ = 81%; n = 3271) and a bacterial meningitis prevalence estimate of 0.4% (95% CI, 0.2%-1.0%; *I*^2^ = 33%; n = 2502). These estimates were similar to those of the full analysis.

## Discussion

We present robust and largely homogeneous pooled prevalence estimates for bacteremia and bacterial meningitis in neonates and infants in the second month of life with fever presenting for ambulatory evaluation. We are unaware of large, previously published data estimating these rates. Health care professionals may use these data as reasonable estimates of pretest probability during clinical decision making. Accurate estimates of pretest probability are necessary for optimal performance of clinical prediction rules and risk calculators.

In the cohort of febrile neonates, our subanalysis of studies in the era after GBS prophylaxis (Figure 3) did not produce different estimates of prevalence but did resolve the excess heterogeneity observed in the after-GBS neonatal bacteremia cohort when compared with the entire cohort of neonatal bacteremia studies (*I*^2^ = 6% vs 50%, respectively). Thus, the pooled prevalence from this subanalysis is more likely to accurately reflect a sample of modern neonates with fever as well as the current risk of bacteremia in this population. The heterogeneity observed in the neonatal meningitis analysis was relatively low in the overall neonatal meningitis cohort and the after-GBS prophylaxis cohort (*I*^2^ = 27% and 28%, respectively).

Similarly, although the data are somewhat older, the bacterial meningitis analysis of infants with fever in the second month of life did not show excess heterogeneity (*I*^2^ = 33%). Therefore, we suggest that this analysis is more likely to reflect the true prevalence of bacterial meningitis in this population.

The analysis of bacteremia in febrile infants in the second month of life showed substantial heterogeneity (*I*^2^ = 78%) that we were unable to resolve through sensitivity analyses by risk. Although our analysis may provide the most thorough prevalence estimate available, the potential inconsistencies within the data reduce the confidence we can ascribe to them, rendering us unable to suggest that these data accurately reflect current rates of bacteremia in this population.^24^

With regard to the clinical evaluation of febrile neonates and infants, presentation in the first vs the second month of life often serves as a branch point used by clinicians to decide whether or not to perform a lumbar puncture or to hospitalize the patient or both.^1,5^ Our pooled prevalence estimate of the rate of bacterial meningitis in febrile neonates in the era after GBS prophylaxis was 1.0%, whereas the estimate in febrile infants in the second month of life was 0.4%. Although this outcome is roughly twice the rate of meningitis in the younger age group, it is unclear whether such a difference in pretest probability obviates the need to perform a lumbar puncture in older infants. The absolute risk difference of 4 per 1000 translates to an extra 250 lumbar punctures per case of meningitis in older infants.

Implicit in our study is the assumption that risk of bacteremia or bacterial meningitis decreases around 30 days of age, and our data support such an assessment. Comparing our prevalence estimates for bacteremia and meningitis by age subgroup suggests that this distinction may be relevant. However, we a priori adopted this age division for our analyses because this age stratification has served for decades as a commonly used clinical standard for decision making.^1,4,5,23,25^ There may be a more meaningful age stratification (eg, <7 days of life); however, the paucity of data does not currently allow for robust estimates to be made outside of the cut point of 1 month of age.^1^

Only 6 of the 12 included studies attempted, in some way, to control for risk of bacterial infection. Thus, although the clinical diagnostic and management conundrums for physicians are often with regard to well-appearing febrile infants, our analysis includes some ill-appearing infants, creating the potential for our pooled analysis to overestimate prevalence rates in well-appearing febrile infants. We can be somewhat reassured, however, that our sensitivity analysis excluding papers that did not provide any attempt to adjust for risk did not identify a significant change in our prevalence estimates (defined by wide overlap in the confidence intervals). That said, we suggest that our prevalence estimates be considered a “ceiling” when it comes to well-appearing febrile infants rather than be considered a true assessment of risk in that population.

### Strengths and Limitations

Our study has several strengths. First, the quality of the included papers for our outcomes of interest was high. Second, a rigorous no-filter search approach, responses to personal communications with a number of corresponding authors, and inclusion of studies examining only consecutive febrile neonates or infants enabled us to provide relatively precise pooled prevalence estimates. Third, our outcomes of interest—positive blood cultures and CSF cultures—function as objective criterion standard measures of bacteremia and bacterial meningitis.

Our study also has limitations. The majority of articles included in this review did not use “fever without a source” as a specific inclusion criterion, and it is possible that this factor resulted in an overestimate of prevalence in this population. The vast majority of studies were performed in the emergency department; therefore, our estimates may not be representative of other clinical areas (eg, outpatient clinics). Regarding the before- and after-GBS prophylaxis subanalysis, it is unclear what, if any, uptake the United States–based recommendation for antibiotic prophylaxis had internationally, particularly regarding data from other countries included in our analysis. Finally, we identified no studies meeting our criteria published within the last 5 years. It is unclear how, or whether, current prevalence rates might differ from those of the published data.

## Conclusions

The results of this systematic review and meta-analysis suggest that the rates of bacteremia and meningitis in febrile neonates are about twice that of infants in the second month of life.

## Notes

Supplement. **eAppendix.** PubMed Search Strategy **eTable.** Newcastle-Ottawa Quality Assessment Scale Scores
