# Supplementary material for: Prevalence of Bacteremia and Bacterial Meningitis in Febrile Neonates and Infants in the Second Month of Life: A Systematic Review and Meta-analysis
Source: JAMA Netw Open. 2019 Mar 22;2(3):e190874. doi: 10.1001/jamanetworkopen.2019.0874 (PMC6583289; doi:10.1001/jamanetworkopen.2019.0874)
Supplement: Supplement. — eAppendix. PubMed Search Strategy eTable. Newcastle-Ottawa Quality Assessment Scale Scores [file jamanetwopen-2-e190874-s001.pdf]

Supplementary Online Content

Biondi EA, Lee B, Ralston SL, et al. Prevalence of bacteremia and bacterial meningitis in febrile neonates and infants in the second month of life: a systematic review and meta-analysis. *JAMA Netw Open*. 2019;2(3):e190874. doi:10.1001/jamanetworkopen.2019.0874

eAppendix. PubMed Search Strategy

eTable. Newcastle-Ottawa Quality Assessment Scale Scores

This supplementary material has been provided by the authors to give readers additional information about their work.

© 2019 Biondi EA et al. *JAMA Network Open*.

eAppendix. PubMed Search Strategy

Search **((((((fever OR fevers OR feverish OR febril* OR febricity OR pyrexi*)) AND ((((((sepsis OR septicemia OR septicaemia)) OR (urinary tract infections OR UTI OR urinary tract infection*)) OR (meningitis, bacterial OR bacteria* OR listeria* OR escherichia OR haemophilus OR hemophilus OR meningococc* OR pneumococc*)) OR (bacteremia OR bacteraemia)) OR (SBI OR SBIs OR serious bacterial infection* OR severe bacterial infection*)))) AND infant[MeSH])) OR (((infant OR infants OR newborn* OR neonat*)) AND (((fever OR fevers OR feverish OR febril* OR febricity OR pyrexi*)) AND ((((((sepsis OR septicemia OR septicaemia)) OR (urinary tract infections OR UTI OR urinary tract infection*)) OR (meningitis, bacterial OR bacteria* OR listeria* OR escherichia OR haemophilus OR hemophilus OR meningococc* OR pneumococc*)) OR (bacteremia OR bacteraemia)) OR (SBI OR SBIs OR serious bacterial infection* OR severe bacterial infection*))))**

© 2019 Biondi EA et al. *JAMA Network Open*.

eTable. Newcastle-Ottawa Quality Assessment Scale Scores

Study	Number of Stars in Each Category			Total
	Selection (max = 4)	*Comparability (max = 2)*[a]	*Outcome (max = 3)*	
Ashkenazi-Hoffnung	4	2	3	9
Bachur and Harper, 2001	3	2	3	8
Baker and Bell, 1999	4	1[b]	3	8
Bonadio et al, 1993	4	2	3	9
Bonsu et al, 2012	3	1[b]	3	7
Caviness et al, 2008	2	1[b]	3	6
Chiu et al, 1994	4	2	3	9
Chiu et al, 1997	4	2	3	9
Ferrera et al, 1997	3	2	3	8
Garcia et al, 2012	3	2	3	8
Herr et al, 2001	3	2	3	8
Zarkesh et al, 2011	3	2	3	8

[a] One point given for an attempt to control for risk above the general febrile infant population (e.g. excluding infants with chronic illness) and one point given for an attempt to control for false positive/false negative culture results (e.g. excluding contaminants)

[b] Accounted for contaminants, but not for risk status (e.g. prematurity, chronic disease, etc…)

© 2019 Biondi EA et al. *JAMA Network Open*.
